# Could outcomes of intracranial aneurysms be better predict using serum creatinine and glomerular filtration rate?

**DOI:** 10.1590/acb370107

**Published:** 2022-04-08

**Authors:** Nícollas Nunes Rabelo, Leonardo Zumerkorn Pipek, Rafaela Farias Vidigal Nascimento, João Paulo Mota Telles, Natalia Camargo Barbato, Antônio Carlos Samaia da Silva Coelho, Guilherme Bitencourt Barbosa, Marcia Harumy Yoshikawa, Manoel Jacobsen Teixeira, Eberval Gadelha Figueiredo

**Affiliations:** 1 PhD. Universidade de São Paulo – Department of Neurosurgery – São Paulo (SP), Brazil.; 2 Graduate student. Universidade de São Paulo – Faculdade de Medicina – São Paulo (SP), Brazil.; 3 Graduate student. Faculdade de Medicina do ABC – Centro Universitário Saúde ABC – Santo André (SP), Brazil.

**Keywords:** Creatinine, Intracranial Aneurysm, Glomerular Filtration Rate

## Abstract

**Purpose:**

To analyze the role of serum creatinine levels as a biomarker of intracranial aneurysm outcomes.

**Methods:**

This is a prospective analysis of outcomes of patients with intracranial aneurysm. One hundred forty-seven patients with serum creatinine at admission and 6 months follow up were included. Linear and logistic regressions were used to analyze the data. Modified Rankin scale (mRS) was used to assess outcome.

**Results:**

Creatinine level was not directly related to aneurysm outcome nor aneurysm rupture (p > 0.05). However, patients with a glomerular filtration rate (GFR) lower than 72.50 mL·min^–1^ had an odds ratio (OR) of 3.049 (p = 0.006) for worse outcome. Similarly, aneurysm rupture had an OR of 2.957 (p = 0.014) for worse outcomes. Stepwise selection model selected 4 variables for outcomes prediction: serum creatinine, sex, hypertension and treatment. Hypertensive patients had, on average, an increase in 0.588 in mRS (p = 0.022), while treatment with microsurgery had a decrease in 0.555 (p = 0.038).

**Conclusions:**

Patients with higher GFR had better outcomes after 6 months. Patients with higher GFR had better outcomes after 6 months. Creatinine presented an indirect role in GFR values and should be included in models for outcome prediction.

## Introduction

Subarachnoid hemorrhage (SAH) cases represent 3% of the causes of stroke, with high rates of mortality and morbidity^1^. The main cause of spontaneous SAH is the rupture of saccular aneurysms, known as aneurysmal subarachnoid hemorrhage (aSAH)^1^ Other less prevalent causes are arteriovenous malformations, fistulas, vasculitis, intracranial arterial dissections and drugs.

Creatinine is known for its role as a marker of renal function, but it is also related to several other factors, including nutritional status^2^ and a biomarker in several pathological processes as an indicator of severity or prognosis. Studies of neurological diseases show a clear correlation between creatinine levels and disease progression, including spinal muscular atrophy^3^^,^ 4, spinal and bulbar muscle atrophy^4^, Duchenne and Becker muscular dystrophy^5^ and acute encephalopathy^6^. Studies in the field of cardiology have also shown the importance of this biomarker in cardiogenic shock^7^, heart failure^8^ and cardiothoracic surgery^9^. Finally, this association has also been demonstrated in other areas, such as oncology^10^^-^ 12, gynecology^13^ and gastrointestinal^14^^,^ 15.

Thus, the present study aims to evaluate the relationship between serum creatinine, glomerular filtration rate (GFR) and long-term outcome after treatment of intracranial aneurysm.

## Methods

### Ethical standards

This research project was approved by the Ethics and Research Committee of the Hospital das Clínicas of FMUSP. Online registration CAPPesq: 15226 approved 06/20/2016. Approved on the Brazil platform CAAE number: 61719416.6.0000.0068. Patient consent was obtained for all participants.

### Study design

This is a prospective cohort study with patients who were admitted in the hospital due to SAH, between January 2018 and November 2019. Social and demographic data from charts of patients from database from the Department of Neurosurgery of the Hospital das Clínicas (HCFMUSP) were collected. Serum creatinine and aneurysm intracranial rupture status upon admission and the modified Rankin scale (mRS) at 6 months were also obtained.

### Population data

**Figure 1:**
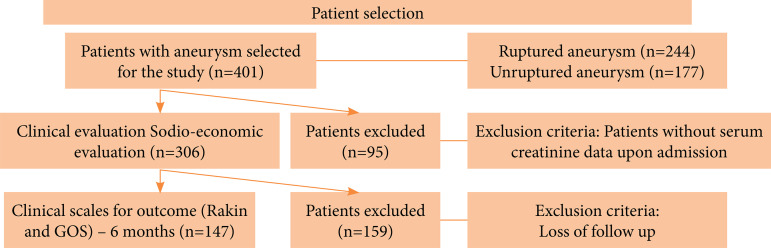
Population data and selection process based on inclusion and exclusion criteria; 147 patients were included in this study.

During this period, 401 patients (adult men and women) were admitted with intracranial aneurysm diagnosis at Hospital das Clínicas da FMUSP (HCFMUSP) Department of Neurological Surgery. From those 401 patients, 147 were included in this study (Fig. 1).

Patients underwent clinical evaluation, which comprised age, sex and race. A questionnaire concerning previous risk factors to aneurysmatic disease was performed, including hypertension, diabetes mellitus, smoking, alcoholism, drug abuse, family history, previous SAH and time from the last event.

Besides that, a socioeconomic evaluation of the participants was performed, assessing scholarship, family income, occupation, and marital status. Based on clinical and imaging conditions, patients were treated with embolization or microsurgery.

Serum creatinine was obtained at admission and patients were followed for 6 months. At the end of the study, mRS was used to measure outcome after SAH.

### Exclusion criteria

Patients without serum creatinine data upon admission or loss of follow up in less than 6 months.

### Inclusion criteria

Patients of both gender and ages, with ruptured and unruptured brain aneurysm who were admitted to the HCFMUSP between January 2018 and November 2019 were included.

### Statistical analysis

Linear and logistic regressions with serum creatine were used as independent and continuous variable. The outcome was quantified by mRS at 6 months. Significance level was established as 0.05. For the logistic regression, unfavorable outcome was defined as mRS > 2.

Cutoff values for GFR were calculated based on receiver operating characteristic (ROC) curves, maximizing the metric function to determine the optimal cut point.

The variable selection method used was stepwise approach. The variables analyzed were serum creatinine, GFR, age, history of previous SAH, gender, age, smoking, alcoholism, aneurysm treatment (embolization or microsurgery) and aneurysm rupture. As unruptured aneurysms were included, Hunt Hess was not considered in the model. Predictor variables were either dropped or added to the logistic regression based on Akaike information criterion. The variables chosen were included in a separate linear regression model (Table 1, model 3).

**Table 1:** Linear regression model for prediction of outcome 6 months after intracranial aneurysm event.

Model 1					
Variables	Ruptured			Unruptured	
	Estimate	p-value		Estimate	p-value
Intercept	3.058	-		2.628	-
Serum creatinine	0.039	0.563		–0.203	0.442
**Model 2**					
**Coefficients:**	**Estimate**			**p-value**	
Intercept	1.076			-	
GFR (> 72.5 mL·min^–1^)	–1.115			0.006*	
Aneurysm rupture	1.084			0.014*	
**Model 3**					
**Coefficients:**	**Estimate**			**p-value**	
Intercept	1.331			-	
Serum creatinine	0.052			0.146	
Gender (female)	0.429			0.085	
Hypertension	0.588			0.022*	
Treatment (microsurgery)	–0.555			0.038*	
**Model 4**					
**Coefficients:**	**Estimate**			**p-value**	
Intercept	0.415			-	
Serum creatinine	0.058			0.180	
GFR	0.002			0.762	
Smoking	0.345			0.149	
Alcoholism	–0.284			0.339	
Gender(female)	0.372			0.160	
Hypertension	0.523			0.050*	
Age	0.011			0.237	
Aneurysm Rupture	0.191			0.492	
Treatment (microsurgery)	–0.579			0.036*	

Model 1: Simple linear regression with serum creatinine as independent variable. Model 2: Logistic regression with GFR and aneurysm rupture as independent variables. Model 3: Multiple linear regression (stepwise) with serum creatinine, sex, hypertension and treatment as independent variables. Model 4: Multiple linear regression (stepwise) with serum creatinine, GFR, smoking, alcoholism, sex, hypertension, age, aneurysm rupture and treatment as independent variables.

Patients were also divided in ruptured and unruptured aneurysm groups. Serum creatinine was analyzed with Welch two sample t-test. The mRS – 6 months was analyzed with Fisher’s exact test for count data. A significance level of 0.05 was used.

The analyses were performed using the statistical software RStudio Team (2015). RStudio: Integrated Development for R. RStudio, Inc., Boston, MA.

## Results

### Epidemiology and comorbidity

**Figure 2:**
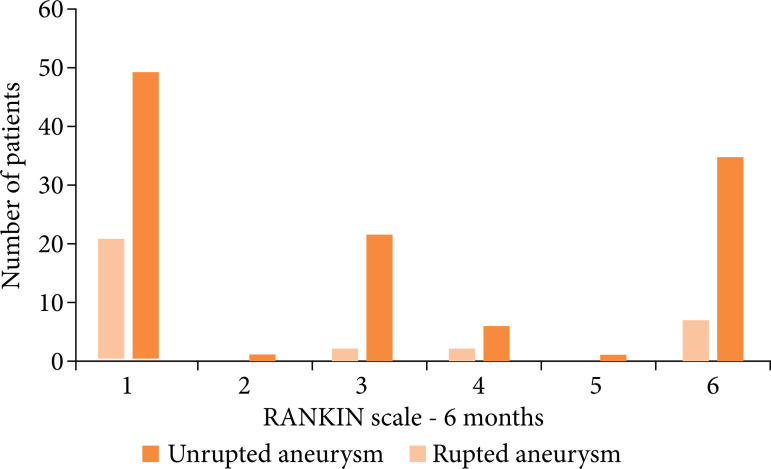
Distribution of ruptured and unruptured aneurysm based on RANKIN – 6 months.

Among the 147 patients included in the study, the average age was 57.06 ± 12.70 years, and 74.2% were female. Hypertension was present in 73.3% of the patients, 37.1% had previous diabetes mellitus, 49.5% were smokers, 20.0% were heavy alcohol drinkers, 14.2% have had SAH previously and 78.2% patients had ruptured aneurysm. Figure 2 shows the distribution of aneurysm rupture and mRS score after 6 months; 73.3% of patients were treated with microsurgery and the others with embolization. The median for the Glasgow coma scale (GCS) at admission was 14. (Table 2).

**Table 2:** Patients’ characteristics. Patients were divided in ruptured and unruptured aneurysm. p-value shows comparison between groups.

Intracranial Aneurysm		Ruptured (115)	Unruptured (32)	p-value
Epidemiology				
	Age (years)	56.76 (12.98)	58.15 (11.09)	0.548
	Gender (male)	82 (71.3%)	28 (87.5%)	0.068
	Hypertension	63 (78.8%)	14 (56.75%)	0.037*
	Diabetes mellitus	30 (37.5%)	9 (36.0%)	1.000
	Smoking	45 (56.2%)	7 (28.0%)	0.021*
	Alcoholism	18 (22.5%)	3 (12.0%)	0.391
	Previous SAH	13 (16.2%)	2 (8.0%)	0.513
	Multiple aneurysm	16 (14.4%)	9 (90.0%)	0.000*
**Clinical scales**				
	Hunt Hess – admission	2(1.22)	-	
	WFNS – admission	2 (1.61)	-	
	GCS – admission	14 (4.26)	-	
	Rankin – 6 months	3 (2.15)	1 (2.11)	0.105
**Variables**				
	Treatment (microsurgery)	75 (68.8%)	27 (90.0%)	0.020*
	GFR (mL·min^–1^)	89.06 (25.28)	81.22 (25.58)	0.130
	Serum creatinine (mg·dL^–1^)	1.19 (3.01)	1.09 (1.46)	0.801

Data is presented as mean (SD) for continuous variables, median (SD) for ordinal variables and count (valid percentage) for categorical variables. SAH: subarachnoid hemorrhage; GCS: admission Glasgow coma scale; GFR: glomerular filtration rate (CKD-EPI).

### Creatinine, GFR and aneurysm rupture

Serum creatinine was measured at hospital admission and was used to test whether it was correlated with aneurysm rupture. Mean serum creatinine in patients with unruptured aneurysm was 1.09, while patients with ruptured aneurysm had a mean creatinine of 1.18. This difference was not statistically significant (p > 0.05). Similarly, mean GFR was not significant different (p > 0.05) in patients with unruptured aneurysm was (81.22 mL·min^–1^) and ruptured aneurysm (89.06) (Table 2).

### Creatinine and outcome

The median mRS at 6 months for all patients included in the study was 3. Linear regression analysis using serum creatinine as a predictor and the mRS at 6 months as outcome shows that serum creatine does not have a statistically significant influence (p > 0.05). Moreover, the subgroup analyses with ruptured and unruptured aneurysm shows no difference in neither group (p > 0.05). (Table 1, model 1). The linear regression with serum creatinine, aneurysm rupture and an interaction term (serum creatinine: aneurysm rupture) did not show any statistically significant variable.

### Glomerular filtration rate and outcome

**Figure 3:**
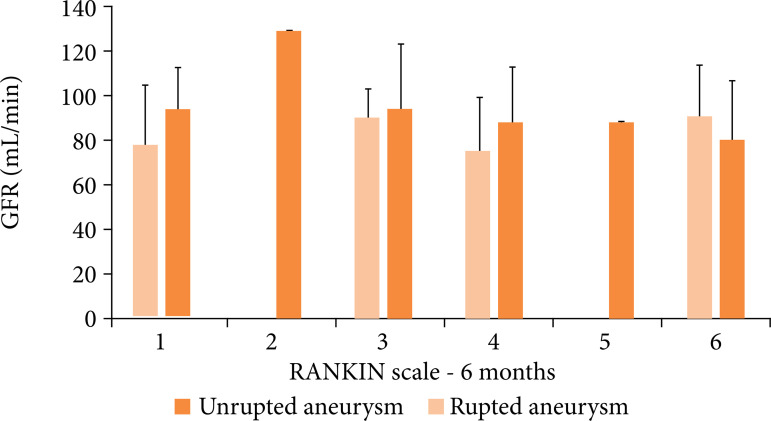
Patients’ admission GFR and Rankin scale at 6 months.

Glomerular filtration rate calculated by CKD-EPI was used to predict outcome measured by mRS at 6 months (Fig. 3). A ROC curve was used to determine the best cutoff point for GFR, 72.50 mL·min^–1^. Logistic regression using GFR and aneurysm rupture as dichotomous variable shows that both of them were significant. Patients with GFR < 72.50 had an OR of 3.049 [1.361–6.833] for worse mRS scores (p = 0.006). Similarly, aneurysm rupture had an OR of 2.957 [1.241–7.043] for worse mRS scores (p = 0.014) (Table 1).

### Stepwise selection model and outcome

The selected variables based on the stepwise selection method were serum creatinine, sex, hypertension and method of treatment (microsurgery or embolization). The model with these variables is represented in Table 1, model 3. These four variables are considered important to build a model for outcome prediction. However, only hypertension (0.588 increase in mean mRS outcome, p = 0.022) and treatment (0.555 decreased mean mRS with microsurgery when compared to embolization, p = 0.038) were individually statistically significant predictors.

Model 4 (Table 1) uses variables significant in other models and the most common variables described in the literature as potential factor that can predict aneurysm outcomes. Hypertension and treatment (microsurgery) were statistically significant predictors (p = 0.050 and 0.036, respectively).

## Discussion

Symptoms of SAH include sudden severe headache that is usually associated with consciousness loss^16^. It can cause secondary vasospasm^17^^,^ 18, which may lead to cerebral ischemia, persistent headache, mental dullness and other severe conditions^19^^-^ 22. Therefore, it is important to identify factors that can early predict aneurysms prior to rupture and, more importantly, predict the long-term outcomes of these patients.

Risk factors such as smoking^23^, alcohol consumption^24^, hypertension^24^^-^ 26, female gender^27^^,^ 28 and age over 50 have been associated with increased chance of aneurysm^29^^-^ 31. This study findings corroborate the association between hypertension and smoking with the rupture of aneurysms causing SAH. Furthermore, those characteristics are not enough to precisely predict neither rupture of IA, nor prognosis of the disease. In these contexts, new biomarkers for intracranial aneurysm have been studied.

MPO, GM-CSF, MCP-1 and other cytokines are cited in numerous studies as potential biomarkers, because of its correlation with inflammation, especially with neutrophils and macrophages^32^^-^ 37. Hostettler *et al*.^38^ found that procalcitonin on days 1, 3, and 7 could be used as prediction variable. The field of machine learning is quickly developing and trying to develop models to predict long term outcomes for intracranial aneurysm^38^^-^ 45. Each study has a slightly different approach to select variables that might have an influence, with some of them being present almost invariably, including age, gender and clinical scales. The relatively small number of patients and absence of all variables available for the same population limits the precision for prediction for those studies.

### Creatinine and GFR

Creatinine is an important biomarker in several diseases. This is most related with muscular denervation, because creatinine plays an important role in muscle fibers homeostasis^46^. In muscular dystrophies^3^^,^ 4 and neurodegenerative disorders^3^^,^ 5, for example, creatinine predicts the outcome because it measures the muscle mass. However, no relation with serum creatinine was found when studying Parkinson’s disease, mainly because it does not involve motor neurons^3^.

When measuring serum creatinine, one is also indirectly assessing creatinine clearance and estimating GFR, which can be calculated from CKD-EPI, and this formula uses serum creatinine values and other variables, including age, gender and race to estimate the outcome of the patients^47^. The correlation between GFR and cardiovascular diseases is already widely discussed in the literature, this relationship occurs because it predicts that the renal excretion is failing, thus increasing the cardiac output and therefore problems related to the cardiovascular system.

Huang *et al*.^31^ found that increased albuminuria was related to higher risk of stroke. The literature reveals that albuminuria is closely related to lower GFR^48^^,^ 49, so one can conclude that lower GFR can be a risk factor for stroke^31^^,^ 46. It is also associated with reduced ejection fraction, which increases the risk for several diseases, such as heart failure^50^. Furthermore, GFR calculated by CKD-EPI is also an important risk factor for bleeding in patients with atrial fibrillation, and, because of that, it can predict the prognosis of these patients^51^.

In this study, patients were divided using a GFR cutoff point of 72.50 (mL·min^–1^). Higher GFR was correlated with lower 6 months Rankin scores. Those patients had an OR of 0.327 for unfavorable outcomes. As previously shown^52^, it was found that ruptured aneurysm was associated with poor outcomes, with an OR of 2.957. The relatively small number of patients with unruptured aneurysm and high Rankin score might be responsible for the lack of statistical significance for creatinine. The study of the role of creatinine and GFR in the pathophysiology of the aneurysms^53^^-^ 55 and others biomarkers can also contribute to clarify this relation, but more research is needed in this field.

A model using stepwise approach that selected the best variables to predict a more precise outcome of the patients in 6 months was used. Although not all selected variables were significant, the method found that creatinine, sex, hypertension and treatment option are the most sensitive to predict the final result. The patients with elevated serum creatinine, presence of hypertension or female gender had higher Rankin scale values when evaluated after 6 months, showing a worsening in the prognosis. On other hand, the multiple linear regression suggests that patients undergoing microsurgery had lower mRS results when compared to patients undergoing embolization, suggesting a better prognosis.

Finally, a model with several variables that have already been described in the literature with a possible association with the outcome of patients with aneurysm was used, in which it was noticed that the GFR loses importance when being in the same model as serum creatinine, gender and age, probably because they are correlated through the formula that were used to calculate the GFR^56^. This model also confirmed the relevance of hypertension and the treatment choice, remaining statistically significant to predict more precisely the outcome based in the mRS of these patients in 6 months.

Future studies should try to elucidate the different mechanism involved in the pathophysiology of intracranial aneurysm, especially the molecular basis for other biomarkers. It is important to prevent and provide better treatment for intracranial aneurysm, but also understand why some patients have better long-term outcomes.

There are some limitations in this study, a clinical trial with randomization of treatment and a longitudinal control of serum creatinine levels is necessary for a clear answer. The inclusion of more variables in the prediction model would demand more patients.

## Conclusion

Models to predict aneurysm outcome are important to take medical decisions that will improve patient’s quality of life. This study shows that creatinine was not directly related to mRS 6 months outcome, but might have a role in predictions models as shown by the stepwise selection model approach. Furthermore, GFR can be used to help predict long term outcomes. Patients with GFR lower than 72.5 mL·min^–1^ had an OR of 3.049 (p = 0.006) for worse outcome after 6 months measured by mRS.
